# Irreducible Fifth Metatarsophalangeal Joint after Car Crush Injury

**DOI:** 10.1155/2015/894057

**Published:** 2015-03-15

**Authors:** Fatih Turkmensoy, Samet Erinc, Omer Naci Ergin, Korhan Ozkan, Bahattin Kemah

**Affiliations:** ^1^Clinic of Orthopaedics and Traumatology, Afyonkarahisar Dinar State Hospital, Turkish Ministry of Health, Turkey; ^2^Department of Orthopaedics and Traumatology, Istanbul Medeniyet University Goztepe Training and Research Hospital, Turkey; ^3^Department of Orthopaedics and Traumatology, Istanbul Faculty of Medicine, Istanbul University, Turkey

## Abstract

Metatarsophalangeal joint dislocations are uncommon injuries. Herein, an irreducible dislocation of fifth metatarsophalangeal joint with fractures on the second, third, and fourth metatarsal head was reported. Joint reduction could not be achieved which necessitated open reduction. Six months after surgery the patient was walking and doing his daily activities without any complaints. He had returned to his pretrauma functional level.

## 1. Introduction 

Although metatarsophalangeal (MTP) joints of the foot are small, they are very stable, and dislocations occur only very rarely [1, 2]. However, if left untreated or not properly managed, a dislocation in this area can have a deleterious effect on the ability of the patient to bear weight and walk [1].

In this report, we present a case of an irreducible fifth MTP joint dislocation with associated fractures of the second, third, and fourth metatarsals to emphasise the importance of a careful physical examination and assessment of the X-ray with proper management.

## 2. Case History

**Figure 1:**
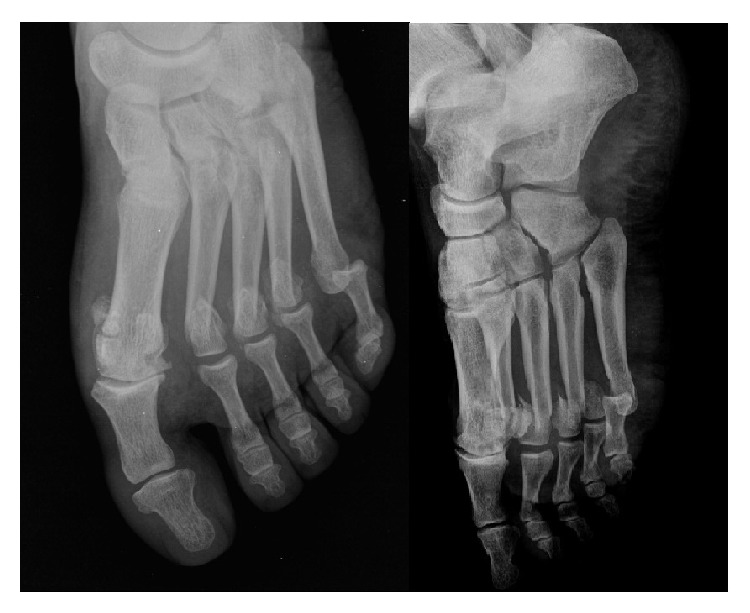
The X-ray of the patient on first examination.

A 66-year-old man was hit by a car as a pedestrian. On admission, his consciousness was clear, but he had pain on the lateral side of his left knee and forefoot. The car had crushed his back and he had fallen down on his left side. Imaging was performed after an initial examination. The X-ray demonstrated fractures on the second, third, and fourth metatarsal heads and the proximal fibula with dislocation of the fifth MTP joint (Figure 1).

Manipulative reduction was performed immediately with local anaesthesia, but it failed. Therefore, he was admitted to the clinic for reduction under regional or general anaesthesia with fluoroscopy. Although manipulative reduction was performed under general anaesthesia, no reduction was achieved. Therefore, we decided on an open reduction due to suspicion of soft tissue entrapment.

**Figure 2:**
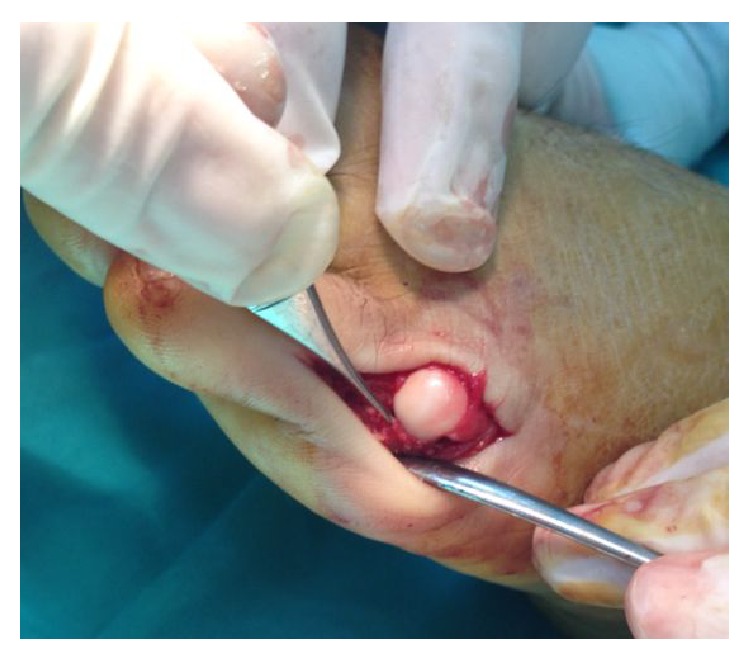
Extracting of entrapped plantar plate from MTP joint.

**Figure 3:**
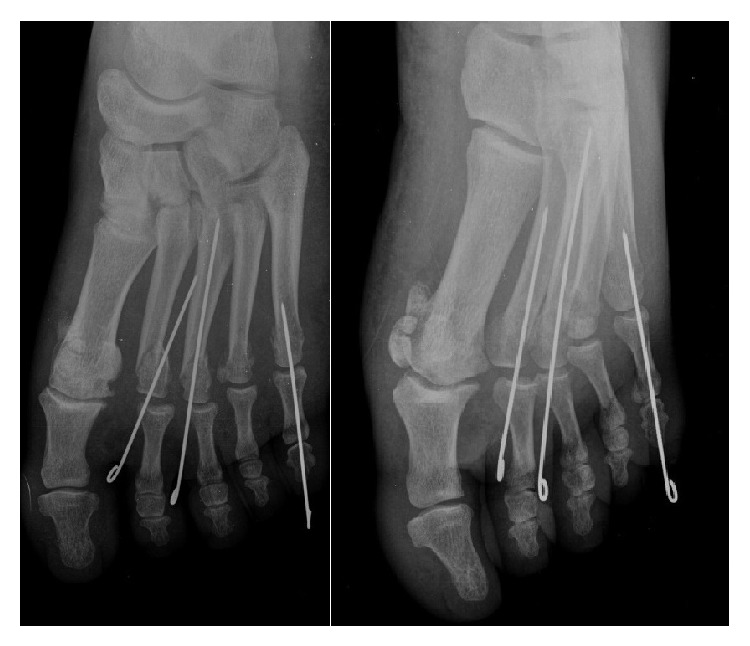
Stabilized dislocated fifth MTP and displaced second and third metatarsal head with transarticular K-wire.

A dorsal approach was used for the open reduction. The plantar plate was turned from its proximal attachment and wedged dorsal to the exposed metatarsal head when the joint capsule was incised transversely. A short longitudinal incision was made to split the volar plate, and the volar plate was seen entrapped in joint and it is easily extracted (Figure 2). The reduction and the displaced second and third metatarsal head were stabilised with a transarticular K-wire (Figure 3).

The patient was discharged on postoperative day 5. After the K-wire was removed, controlled weight-bearing was initiated on postoperative week 6, and full weight-bearing was achieved on postoperative week 8. He had no complaints of pain after long-term walking 6 months after surgery.

## 3. Discussion

The medial, lateral collateral, and dorsal and plantar ligaments are the main stabilisers of the MTP joints. Intermetatarsal ligaments, interosseous tendons, lumbricals, flexor tendons, and the extensor digitorum longus and brevis also provide additional support.

Although the MTP joint is small, it is very stable and dislocations occur very rarely [1–4]. Irreducible dislocations are the only reported case reports in the English literature. Dislocations are usually dorsal in direction, but horizontal and plantar dislocations have also been reported [5]. These dislocations usually occur due to high energy dorsiflexion and axial loading forces. This type of forceful dorsiflexion and axial loading can cause additional fractures and dislocations of the foot [5]. Dorsiflexion and axial loading caused a fifth MTP joint dislocation and a second, third, and fourth metatarsal head fracture after the fall, which was most likely the injury mechanism in this case.

The plantar plate incorporates the plantar aponeurosis and the plantar capsule of the MTP joint. The main function of this strong and longitudinally oriented structure is to resist pressure by the metatarsal head. In addition to this absorbent function, it also lubricates joints and flexor tendons [2, 6]. The plantar plate shows some similarities with the volar plate of the hand, as both structures are the main reason for irreducible metacarpophalangeal versus MTP joint dislocations [7].

Reduction was achieved easily in the present case when the plantar plate was removed from the MTP joint. However, the MTP joint was stabilised temporarily with a K-wire to provide healing of the plantar plate.

MTP joint dislocations may easily be missed, particularly in the presence of other injuries. Careful evaluation of the patient and proper management are required to prevent possible lifelong disability due to the small-sized but the functionally important MTP joint.
